# Persistence of Racial and Ethnic Disparities in Adverse Maternal and Birth Outcomes Among Physicians

**DOI:** 10.1097/og9.0000000000000086

**Published:** 2025-06-24

**Authors:** Michelle Ann Caesar, Rebecca Baer, Gretchen Bandoli

**Affiliations:** Herbert Wertheim School of Public Health and Human Longevity Science and the Department of Pediatrics, School of Medicine, University of California, San Diego, La Jolla, California and the University of California, San Francisco, San Francisco, California.

## Abstract

**OBJECTIVE:**

To evaluate whether racial disparities persisted among physicians giving birth, to equalize individual sociodemographic factors.

**METHODS:**

Singleton live births among California physicians between 2012 and 2019 were linked to pregnancy and delivery hospitalization records. Physicians were identified using standardized occupational codes from birth records. Adjusted risk differences (aRDs) by self-reported race and ethnicity were calculated.

**RESULTS:**

Overall, 14,878 physicians in California had live births. Compared with non-Hispanic White physicians, Asian physicians had an increased risk of gestational diabetes (aRD 0.08, 95% CI, 0.05–0.11) and of having neonates with small-for-gestational-age (SGA) birth weight (aRD 0.05, 95% CI, 0.04–0.07). Non-Hispanic Black physicians had an increased risk of having neonates with SGA birth weight (aRD 0.05, 95% CI, 0.02–0.09) and of preterm birth (aRD 0.07, 95% CI, 0.01–0.13), and physicians who identified as additional races/unknown (two or more races, other, or not stated or unknown race) had an increased risk of preeclampsia (aRD 0.07, 95% CI, 0.01–0.12).

**CONCLUSION:**

Racial and ethnic disparities persisted for select perinatal outcomes among California physicians. These results highlight the need to directly assess system-level factors that contribute to these disparities.

## Abstract 2

Racial and ethnic disparities persisted for select perinatal outcomes among California physicians, highlighting the need to directly assess system-level factors that contribute to these disparities.

Previous literature has documented racial and ethnic disparities in adverse pregnancy and birth outcomes.^1–7^ Although there have been advances in medical care and increased attention at the federal level to improve the health of birthing people and neonates, members of certain racial and ethnic groups continue to experience a high rate of pregnancy complications and poor birth outcomes.^1,2,5,6^ Disparities are best documented among non-Hispanic Black individuals. Black individuals are three times more likely to die from a pregnancy-related cause, have a twofold greater risk of preterm birth, are twice as likely to have a birth with late or no prenatal care, and have a higher rate of cesarean delivery compared with White individuals.^2,5,6,8^

These racial and ethnic disparities have also been well documented in California state-wide data.^8–10^ Ratnasiri et al^9^ examined temporal trends and predictors of preterm birth and found that prevalence of preterm birth declined by more than 5.0% between 2007 and 2016, but that the decrease was not equal across racial groups. The prevalence of preterm birth declined the most among White individuals, followed by Black individuals; Hispanic individuals had a slight increase in prevalence (0.1%).^9^ In 2023, the rate of preterm birth in California was highest among individuals who identified as Black (12.7%), followed by American Indian and Alaska Native (11.1%), Hispanic (9.3%), Asian and Pacific Islander (8.9%), and then White (7.7%).^11^ In a separate study of California births between 2007 and 2011, preterm birth complicated 6% of births to White individuals and 10% of births to Black individuals, which was observed across all types of preterm birth (spontaneous and medically indicated).^11^ Similar disparities are also present in other adverse outcomes such as preeclampsia, cesarean delivery, and several maternal morbidity and mortality,^8,12,13^ highlighting the inequity in birth outcomes across multiple racial and ethnic groups in the state.

Several factors likely contribute to these disparities, including variation in the quality and quantity of health care, underlying health conditions, and social determinants of health, particularly those related to the historical and contemporary forms of structural and institutional racism.^2,10,12,14,15^ Many studies,^2,4,14,15^ including studies using California data,^10,12^ have tried to account for socioeconomic differences between races to determine the extent to which disparities remain. However, due to the incomplete capture of sociodemographic and other individual-level variables, racial inequities often remain confounded not only by socioeconomic status (SES) but by factors that cluster with low SES, such as lack of access to quality health care; low health literacy; transportation, childcare, or other logistical barriers; and degree of control over one's employment and job stressors.^16,17^ The control of these individual-level factors would allow one to isolate the disparities that remain, which reasonably could be attributed to racially based factors such as institutional and structural racism. In an effort to equalize individual-level socioeconomic factors and to better isolate remaining structural level racial inequities, the objective of the study was to estimate adverse pregnancy and birth outcomes by race and ethnicity in a sample of practicing physicians who gave birth in the California between 2012 and 2019.

## METHODS

The Study of Outcomes in Mothers and Infants is a retrospective birth cohort comprised of vital statistics files of all births and fetal death records in California from 2012 to 2019. Hospitalization records retrieved from the Department of Healthcare Access and Information were linked to vital statistics records. These hospitalization records provided diagnostic codes based on the International Classification of Diseases, Ninth Revision, Clinical Modification (ICD-9-CM) and Tenth Revision, Clinical Modification (ICD-10-CM) fields. Discharge records were linked to neonatal birth record files for the pregnant person (here termed maternal health records) 1 year before the child’s birth through 1 year after the birth. Additionally, any infant discharge summaries in the year after birth were linked to the dyad.

There were 3,870,488 children born in California between 2012 and 2019, of whom 3,487,610 were singleton live births successfully linked to Department of Healthcare Access and Information maternal and child records. The study was approved by the University of California, San Diego, Human Research Protection Program.

The pregnant person's usual occupation and usual type of business or industry were self-reported as unstructured text in the birth record. We first excluded anyone missing the date last worked before delivery (n=1,171,786; 2,324 of whom identified as physicians) and those whose last day of work did not overlap with pregnancy (n=166,901; 610 of whom identified as physicians; Appendix 1, available online at http://links.lww.com/AOG/E166). In this occupational cohort, these criteria were applied to ensure equal access to health care and resources during pregnancy and the continued physical ability to work. We used the National Institute for Occupational Safety and Health Industry and Occupation Computerized Coding System to classify the unstructured text into standardized occupational codes for all deliveries.^18,19^ From the coded data, we identified observations where the standardized occupational code was assigned a value of 29-1067 or 29-1069 (physicians and surgeons) and exported the raw text value. One author (G.B.) reviewed each record to compare the free text with the designated occupation of physicians. Work in related environments (eg, doctor of chiropractic medicine, staff in medical offices, medical students) were excluded. This process resulted in an analytic cohort of 14,878 individuals who gave birth and identified as physicians.

Exposure, outcomes, and covariates of interests were identified using health records that occurred during the pregnancy or delivery, or from birth record variables. Given the objective to evaluate racial disparities, the primary exposure was self-reported maternal race and ethnicity as captured on the neonatal birth records. Categorization was based on the following: We first coded Hispanic based on any selection of Hispanic (regardless of whether a race option was selected). Then, from individuals who did not identify as Hispanic, we categorized individuals who selected only non-Hispanic White, non-Hispanic Black, Asian, Native American and American Indian, or Hawaiian and Pacific Islander. Individuals who selected two or more races (n=436), other (n=122), or unknown or not stated (n=155) were categorized as additional races/unknown. Finally, due to small numbers, Native American and American Indian (n=72) and Hawaiian and Pacific Islander (n=6) were combined with the additional races/unknown category.

Primary maternal outcomes were identified through health records from any visit during pregnancy, delivery, or up to 6 weeks postpartum (for severe maternal morbidity and mortality). Maternal outcomes of interest included gestational diabetes (ICD-9-CM: 648.8, ICD-10-CM: O24.4; abstracted from birth records and health records), preeclampsia (ICD-9-CM: 642.4–642.7, ICD-10-CM: O14, O15; abstracted from birth records and health records), maternal mortality (ICD-10-CM: A34, O00-O95, O98-O99; from death records), and severe maternal morbidity (abstracted from maternal health records). Severe maternal morbidity, which includes unexpected outcomes of labor and delivery, was defined using the published Centers for Disease Control and Prevention algorithm.^20^ The Centers for Disease Control and Prevention uses 21 indicators to identify delivery hospitalizations with severe maternal morbidity.^20^ These indicators include acute myocardial infarction, aneurysm, acute renal failure, acute respiratory distress syndrome, amniotic fluid embolism, cardiac arrest and ventricular fibrillation, conversion of cardiac rhythm, disseminated intravascular coagulation, blood transfusion, eclampsia, heart failure and arrest during surgery or procedure, puerperal cerebrovascular disorders, pulmonary edema and acute heart failure, severe anesthesia complications, sepsis, shock, sickle cell disease with crisis, air and thrombotic embolism, hysterectomy, temporary tracheostomy, and ventilation.^20^ Primary birth outcomes of interest were identified from the neonatal birth records and included *small-for-gestational-age (SGA) birth weight* (defined as birth weight less than the 10th percentile), *preterm birth* (defined as delivery before 37 weeks of gestation), and neonatal intensive care unit (NICU) admission.

Models of race and ethnicity do not meet traditional definitions of confounding because none of the factors caused race and ethnicity. However, sociodemographic characteristics, exposures, and chronic health conditions are associated with adverse birth outcomes and may vary by race and ethnicity. Thus, the following variables were included as covariates: age at the time of delivery, prepregnancy body mass index (BMI, calculated as weight in kilograms divided by height in meters squared; under or at reference weight [lower than 25], overweight [25–30], or obesity [higher than 30]), source of payment for the delivery (private, public, or other payment source, used as a proxy variable to capture variation in income level), and smoking during pregnancy. These variables were obtained from the neonatal birth record. Additional covariates abstracted from maternal health records during pregnancy or delivery included diagnosis of tobacco use disorder (ICD-9-CM 305.1, 649.0, ICD-10-CM: O99.33, F17), anxiety disorder (ICD-9-CM: 300, ICD-10-CM: F41), and depression (ICD-9-CM: 311, ICD-10-CM: F32-34, F39) each dichotomized as yes or no.

Descriptive statistics for the study population of physicians were calculated by race and ethnicity. Unadjusted prevalence estimates were calculated for each pregnancy complication and birth outcome by race and ethnicity, followed by risk differences (RDs) using generalized linear models. Non-Hispanic White served as the reference group for all multivariable models. Models were adjusted for the age of the person giving birth, prepregnancy BMI, payer source, tobacco use, anxiety disorder diagnosis, and depression diagnosis. Complete case analysis was employed. All analyses were performed using SAS 9.4.

## RESULTS

Between 2012 and 2019, 14,878 singleton children were born to physicians in California. Of these, 42.5% of individuals giving birth were Asian, 40.8% non-Hispanic White, 8.9% Hispanic, 5.3% additional races/unknown, and 3.2% non-Hispanic Black. Across all racial and ethnic groups, mean age at delivery was approximately 35 years, and the majority of individuals had private insurance and could be classified as underweight or at reference weight (Table 1). Overall, fewer than 1.0% used tobacco during pregnancy. Across all racial and ethnic groups, 1.0–2.6% had an anxiety disorder diagnosis and 1.0–3.6% had a depression diagnosis (Table 1). Although less imbalanced than the full California population, differences by race and ethnicity were noted. More Hispanic and non-Hispanic Black physicians had public insurance or other sources of insurance and had overweight or obesity compared with the rest of the sample, and there were more non-Hispanic White physicians with depression or anxiety diagnoses.

**Table 1.** Pregnancy and Health Characteristics of Physicians Giving Birth in California, 2012–2019 (N=14,878)

Characteristic	Race and Ethnicity				
	Asian (n=6,282)	Hispanic (n=1,311)	Non-Hispanic Black (n=467)	Non-Hispanic White (n=6,027)	Additional Races and Ethnicities (n=791)
Maternal age (y)	35.2±3.6	34.6±4.0	35.2±4.3	35.1±3.7	35.1±3.7
Source of payment					
Private insurance	5,390 (85.8)	954 (72.7)	369 (79.0)	5,760 (95.6)	643 (81.2)
Public insurance	88 (1.4)	63 (4.8)	27 (5.8)	94 (1.5)	13 (1.6)
Other	804 (12.8)	294 (22.4)	71 (15.2)	173 (2.9)	135 (17.1)
Missing	0 (0.0)	0 (0.0)	0 (0.0)	0 (0.0)	0 (0.0)
Prepregnancy BMI category					
Underweight or reference weight	5,038 (80.1)	830 (63.3)	263 (56.3)	4,577 (75.9)	592 (74.8)
Overweight	799 (12.8)	325 (24.8)	113 (24.2)	951 (15.8)	125 (15.8)
Obesity	212 (3.4)	132 (10.1)	70 (14.9)	330 (5.5)	51 (6.4)
Missing	233 (3.7)	24 (1.8)	21 (4.5)	169 (2.8)	23 (2.9)
Tobacco use during pregnancy	8 (0.1)	1 (0.0)	0 (0.0)	11 (0.1)	2 (0.3)
Anxiety disorder diagnosis	64 (1.0)	31 (2.4)	7 (1.5)	159 (2.6)	14 (1.8)
Depression diagnosis	64 (1.0)	31 (2.4)	11 (2.4)	214 (3.6)	28 (3.5)
Gestational age (d) at last work day	253.5±33.1	253.1±33.4	248.1±39.9	253.6±33.4	254.9±30.8

BMI, body mass index.

Data are mean±SD or n (column %).

**Fig. 1.**
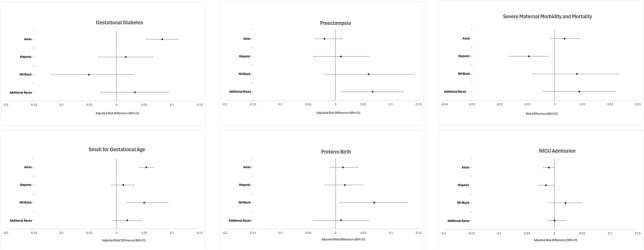
Risk difference by pregnancy complication or birth outcome.

Prevalence of gestational diabetes was highest among Asian physicians, at 14.2%, followed by 10.2% among additional races/unknown physicians, 8.9% among Hispanic physicians, 8.0% among non-Hispanic White physicians, and 6.4% among non-Hispanic Black physicians (Table 2). Compared with White physicians, the adjusted risk difference (aRD) was 8.0% higher for Asian physicians (95% CI, 0.05–0.11), 3.0% higher for additional races/unknown physicians (95% CI, −0.03 to 0.09), and 2.0% higher for Hispanic physicians (95% CI, −0.03 to 0.07), but 5.0% lower for non-Hispanic Black physicians (95% CI, −0.12 to 0.03). However, all groups, with the exception of Asian physicians, had effect estimates that included the null (Table 2 and Fig. 1).

**Table 2.** Unadjusted and Adjusted Risk Differences by Pregnancy Complication*

Complication	Asian (n=6,282)			Hispanic (n=1,311)			Non-Hispanic Black (n=467)			Non-Hispanic White (n=6,027) (Ref)	Additional Races/Unknown (n=791)		
	n (%)	RD (95% CI)	aRD (95% CI)	n (%)	RD (95% CI)	aRD (95% CI)	n (%)	RD (95% CI)	aRD (95% CI)	n (%)	n (%)	RD (95% CI)	aRD (95% CI)
Gestational diabetes	894 (14.2)	0.06 (0.05–0.07)	0.08 (0.05–0.11)	116 (8.9)	0.01 (−0.01 to 0.03)	0.02 (−0.03 to 0.07)	30 (6.4)	−0.02 (−0.04 to 0.01)	−0.05 (−0.12 to 0.03)	484 (8.0)	81 (10.2)	0.03 (0.00–0.05)	0.03 (−0.03 to 0.09)
Preeclampsia	257 (4.1)	−0.01 (−0.01 to 0.00)	−0.02 (−0.04 to 0.01)	64 (4.9)	0.0 (−0.01 to 0.02)	0.01 (−0.03 to 0.05)	44 (9.4)	0.05 (0.02–0.08)	0.06 (−0.02 to 0.14)	280 (4.7)	56 (7.1)	0.02 (0.01–0.04)	0.07 (0.01–0.12)
SMM and mortality^†^	148 (2.4)	0.00 (0.00–0.01)	—	15 (1.1)	−0.01 (−0.02 to 0.00)	—	13 (2.8)	0.01 (−0.01 to 0.02)	—	120 (2.0)	22 (2.8)	0.01 (0.00–0.02)	—

Ref, reference; RD, risk difference; aRD, adjusted risk difference; SMM, severe maternal morbidity.

* Models were adjusted for age, prepregnancy body mass index, payer source, tobacco use, anxiety disorder diagnosis, and depression diagnosis.

†Adjusted model for SMM and mortality did not converge.

*Caesar. Adverse Maternal and Birth Outcomes in Physicians. O&G Open 2025*.

Preeclampsia was highest among non-Hispanic Black physicians, at 9.4%, followed by 7.1% among additional races/unknown physicians, 4.9% among Hispanic physicians, 4.7% among non-Hispanic White physicians, and 4.1% among Asian physicians (Table 2). Compared with non-Hispanic White physicians, the aRDs for preeclampsia were 6.0–7.0% higher among non-Hispanic Black and additional races/unknown physicians (95% CI, −0.02 to 0.14 and 0.01–0.12, respectively) (Table 2 and Fig. 1), although estimates for non-Hispanic Black physicians included the null (Table 2).

Prevalence of severe maternal morbidity was highest among non-Hispanic Black physicians and additional races/unknown, at 2.8%, followed by 2.4% among Asian physicians, 2.0% among non-Hispanic White physicians, and 1.1% among Hispanic physicians (Table 2). Due to sample size, unadjusted RDs were compared for severe maternal morbidity and showed that in comparison with non-Hispanic White physicians, the risk of severe maternal morbidity differed little by any group and all estimates included the null (Table 2 and Fig. 1).

Prevalence of SGA birth weight was highest among Asian physicians, at 14.3%, followed by 12.9% among non-Hispanic Black physicians, 10.9% among additional races/unknown physicians, 9.1% among Hispanic physicians, and 8.9% among non-Hispanic White physicians (Table 3). Compared with non-Hispanic White physicians, aRDs of SGA birth weight were 5.0% higher for neonates of non-Hispanic Black physicians (95% CI, 0.02–0.09) and 5.0% higher for neonates Asian physicians (95% CI, 0.04–0.07) (Table 3 and Fig. 1).

**Table 3.** Unadjusted and Adjusted Risk Differences by Birth Outcome*

Outcome	Asian (n=6,282)			Hispanic (n=1,311)			Non-Hispanic Black (n=467)			Non-Hispanic White (n=6,027) (Ref)	Additional Races/Unknown (n=791)		
	n (%)	RD (95% CI)	aRD (95% CI)	n (%)	RD (95% CI)	aRD (95% CI)	n (%)	RD (95% CI)	aRD (95% CI)	n (%)	n (%)	RD (95% CI)	aRD (95% CI)
SGA birth weight	897 (14.3)	0.05 (0.04–0.07)	0.05 (0.04–0.07)	119 (9.1)	0.00 (−0.02 to 0.02)	0.01 (−0.01 to 0.03)	60 (12.9)	0.04 (0.01–0.07)	0.05 (0.02–0.09)	535 (8.9)	86 (10.9)	0.02 (0.00–0.04)	0.02 (−0.01 to 0.05)
Preterm birth	541 (8.6)	0.01 (0.00–0.02)	0.01 (−0.01–0.04)	107 (8.2)	0.01 (−0.02 to 0.02)	0.02 (−0.02 to 0.05)	65 (13.9)	0.06 (0.02–0.09)	0.07 (0.01–0.13)	489 (8.1)	67 (8.5)	0.00 (−0.02 to 0.02)	0.01 (−0.04 to 0.06)
NICU admission	373 (5.9)	−0.01 (−0.02 to 0.00)	-0.01 (−0.02 to 0.00)	62 (4.7)	−0.02 (−0.03 to −0.01)	−0.02 (−0.03 to 0.00)	41 (8.8)	0.02 (−0.01 to 0.05)	0.02 (0.00 –0.05)	415 (6.9)	55 (7.0)	0.00 (−0.02 to 0.02)	0.00 (−0.01 to 0.02)

Ref, reference; RD, risk difference; aRD, adjusted risk difference; SGA, small for gestation age; NICU, neonatal intensive care unit.

* Models were adjusted for age, prepregnancy body mass index, payer source, tobacco use, anxiety disorder diagnosis, and depression diagnosis.

Prevalence of preterm birth was highest among non-Hispanic Black physicians, at 13.9%, followed by Asian physicians, at 8.6%, and approximately 8.5% among non-Hispanic White, Hispanic, and additional races/unknown physicians (Table 3). Compared with non-Hispanic White physicians, aRDs of preterm birth in was 7.0% higher in non-Hispanic Black physicians (95% CI, 0.01–0.13) and 2.0% higher in Hispanic physicians (95% CI, −0.02 to 0.05), although estimates for the Hispanic physicians included the null (Table 3). There was no difference in the risk of preterm birth between Asian physicians or additional races/unknown physicians and non-Hispanic White physicians (Fig. 1).

Admission to NICU was highest among neonates of non-Hispanic Black physicians (8.8%), followed by 7.0% among neonates of additional races/unknown physicians, 6.9% among non-Hispanic White physicians, 5.9% among Asian physicians, and 4.7% among Hispanic physicians (Table 3). Compared with neonates of non-Hispanic White physicians, aRDs of NICU admission were 2.0% higher among neonates of non-Hispanic Black physicians (95% CI, 0.00–0.05) but 2.0% lower among neonates of Hispanic physicians (95% CI, −0.03 to 0.00); however, these estimates included the null (Table 3 and Fig. 1).

## DISCUSSION

Racial disparities in adverse pregnancy complications have been widely studied. Prior literature has examined how socioeconomic disparities and racism or racial discrimination contribute to adverse outcomes.^7,21^ Our study found that racial and ethnic disparities in certain pregnancy complications and birth outcomes persist in a sample of physicians who represent a group of high socioeconomic and health-literate individuals, suggesting that factors other than sociodemographic characteristics are contributing to these persistent disparities. After adjustment for covariates, non-Hispanic Black physicians generally had the greatest difference in risk of each outcome of interest compared with non-Hispanic White physicians, with the exception of SGA birth weight and gestational diabetes, where Asian physicians had the highest risk. Thus, even among a sample with presumably extremely similar income, education, access to health care and health literacy, racial and ethnic differences persisted.

Although other studies have attempted to isolate nonsocioeconomic contributors to racial disparities, to our knowledge, none have attempted this by conducting the study within a population of physicians. Results of this study of physicians are in accord with McGrady et al who found that in a sample of 3,084 Black and White female college graduates, Black graduates had 1.67 times greater risk of preterm birth than White graduates.^22^ Ross et al^12^ reported that higher SES reduced the risk of preeclampsia among non-Hispanic White individuals; however, higher SES did not have the same effect among non-Hispanic Black individuals. Similarly, we found that even in a sample of physicians with likely similar SES, the risk of preeclampsia was approximately twice as high among non-Hispanic Black physicians than non-Hispanic White physicians. Another study that examined trends in cesarean delivery in California from 2011 to 2017 found that the rate of cesarean delivery decreased for White individuals but not for Black individuals.^23^ Those authors believed that the differential rates in cesarean delivery might contribute to increased rates of maternal morbidity and mortality among Black birthing individuals.^23^ Similarly, our study found risk of severe maternal morbidity remained higher among non-Hispanic Black physicians compared with non-Hispanic White physicians, although cell sizes were too small to perform multivariable adjustment. In this study, we found that risk of NICU admission was higher among neonates of non-Hispanic Black physicians compared with non-Hispanic White physicians; this is in accord with de Jongh et al,^24^ who found that, among privately insured individuals, neonates of Black individuals had increased odds of NICU admission compared with those of White or Hispanic individuals. Interestingly, neonates born to Hispanic physicians were slightly less likely to be admitted to the NICU relative to those born to non-Hispanic White physicians, despite having similar rates of prematurity and SGA birth weight. These results align with a national study conducted over a similar timeframe, warranting future investigation.^25^ Results of this study are also in accord with Wagner et al^26^ who found that birthing people of all Asian ethnic groups had higher rates of gestational diabetes compared with White people giving birth.

Previous literature has reported the association of racism with adverse pregnancy complications and birth outcomes.^8,15,27^ Structural racism, which manifests through mechanisms such as redlining and residential segregation, mass incarceration, police violence, unequal medical care and disproportionate burden of environmental hazards, contribute to poor outcomes.^15,28^ Societal actions are needed to dismantle structural racism, which can include documenting the effect of racism on health outcomes, improving the measurement of racism, reflecting on the history of racism in the United States, and acknowledging that policies are needed to improve the health of people of color.^28^

This study is not without limitations. First, we had no direct measure of discrimination or any other structural factors, but instead attempted to equalize sociodemographic, access and health literacy factors to determine whether disparities persisted. Thus, although we hypothesize that structural factors contribute to our findings, this was not able to be tested in this sample. Additionally, this study lacked direct information on income, but payer source was used as a proxy and practicing physicians within California likely have relatively similar incomes to each other compared with individuals across the state. This study also was unable to examine subgroup differences in those who identify as Native American or American Indian, Hawaiian, and other Pacific Islander due to small sample size. We also aggregated individuals with two or more races and those with unknown race. The additional races/unknown group is, thus, hard to interpret, and better disaggregation in future studies would be important. Finally, we did not have information on medical specialty, which may have modified the estimated risks.

In conclusion, this study found that racial and ethnic disparities persisted for pregnancy complications and birth outcomes among physicians in California highlighting the important influence of broader sociostructural factors on pregnancy and birth outcomes. Further, the results highlight that in addition to the well-documented disparities in adverse birth outcomes of Black individuals, disparities also are present and deserve attention for birthing individuals who are Asian and for those of more than one race or of unknown race.
